# Xist RNA repeat E is essential for ASH2L recruitment to the inactive X and regulates histone modifications and escape gene expression

**DOI:** 10.1371/journal.pgen.1006890

**Published:** 2017-07-07

**Authors:** Minghui Yue, Akiyo Ogawa, Norishige Yamada, John Lalith Charles Richard, Artem Barski, Yuya Ogawa

**Affiliations:** 1 Division of Reproductive Sciences, Perinatal Institute, Cincinnati Children’s Hospital Medical Center, Cincinnati, Ohio, United States of America; 2 Department of Pediatrics, University of Cincinnati College of Medicine, Cincinnati, Ohio, United States of America; 3 Division of Allergy & Immunology and Human Genetics, Cincinnati Children’s Hospital Medical Center, Cincinnati, Ohio, United States of America; University of Pennsylvania, UNITED STATES

## Abstract

Long non-coding RNA Xist plays a crucial role in establishing and maintaining X-chromosome inactivation (XCI) which is a paradigm of long non-coding RNA-mediated gene regulation. *Xist* has Xist-specific repeat elements A-F which are conserved among eutherian mammals, underscoring their functional importance. Here we report that Xist RNA repeat E, a conserved *Xist* repeat element in the *Xist* exon 7, interacts with ASH2L and contributes to maintenance of escape gene expression level on the inactive X-chromosome (Xi) during XCI. The Xist repeat E-deletion mutant female ES cells show the depletion of ASH2L from the Xi upon differentiation. Furthermore, a subset of escape genes exhibits unexpectedly higher expression in the repeat E mutant cells than the cells expressing wildtype *Xist* during X-inactivation, whereas the silencing of X-linked non-escape genes is not affected. We discuss the implications of these results to understand the role of ASH2L and Xist repeat E for histone modifications and escape gene regulation during random X-chromosome inactivation.

## Author summary

Xist long noncoding RNA induces X chromosome inactivation by recruiting multiple chromatin modifying enzymes to the inactive X-chromosome. Among such chromatin modifying enzymes, ASH2L is unique since it is a component of the MLL/SET complexes, which are a histone lysine 4 methyltransferase for transcriptional activation. Among the novel results in our paper, we found that ASH2L binds to the repeat E of Xist RNA. Using the Xist repeat E deletion mutant cells, we discovered that the repeat E of Xist RNA is dispensable for X-linked gene silencing, but is required for maintaining proper level of escape gene expression. This is the first report indicating that Xist RNA engages not only in X-linked gene silencing but also in escape gene regulation.

## Introduction

To equalize the imbalance of X-linked gene dosage between genders, one of the two X chromosomes in female mammals is converted to the transcriptionally inactive X-chromosome (Xi) at the early phase of development whereby the majority of X-linked genes on the Xi are silenced [1–3]. In mice, imprinted X-chromosome inactivation (XCI) starts from as early as 2-4-cell stage in which the paternal X-chromosome is always silenced, and the paternally imprinted Xi is sustained in the cells of the trophectoderm and primitive endoderm, which give rise to the extra-embryonic tissues [4,5]. In contrast, imprinted XCI is reactivate in the epiblast of the inner cell mass, and then induces random XCI during the peri-implantation stage. The paternal and maternal X chromosomes have an equal chance of inactivation in random XCI.

Once random XCI is established in epiblast lineage, the Xi is inherited throughout successive cell divisions to continue balancing X-linked gene dosage between males and females. While the majority of X-linked genes are subjected to silencing by XCI, a subset of genes called escape genes (approximately 3% in mice) is partially transcribed on the Xi [6,7]. Transcriptionally active compartments for escape genes are marked by characteristic epigenetic modifications which are distinct from heterochromatic regions across the Xi. Some escape genes are commonly expressed on the Xi in variable cell lines and tissues, whereas expression of other escape genes occurs in a cell line- or tissue-specific manner. Although several models have been proposed to explain how escape genes evade XCI under the chromosome-wide heterochromatic environment on the Xi [6,8], the underlying mechanism for escaping XCI remains unknown.

The X inactivation center (*Xic*) required for XCI harbors a number of non-protein-coding (non-coding) genes such as *Xist* and its antisense *Tsix* [9–11]. The expression of *Xist* is upregulated at the onset of random XCI by the coordinated action of several non-coding genes in the *Xic*. Xist RNA plays the pivotal role to initiate XCI and induce chromosome-wide X-linked gene silencing across the Xi except for escape genes [12]. *Xist* encodes a ~17 kb-length non-coding RNA and is comprised of seven exons in mice [13]. In undifferentiated mouse embryonic stem (ES) cells, *Xist* is transcribed at a very low level from both paternal and maternal X chromosomes. When random XCI begins upon differentiation, the expression of *Xist* is increased in the future Xi; meanwhile, *Xist* expression is gradually erased from the other X [14]. The abundant Xist RNA covers the future Xi in *cis* and functions as a scaffold for various chromatin modifiers such as polycomb repressive complex 2 (PRC2) and PRC1 for histone 3 lysine 27 trimethylation (H3K27me3) and histone 2A lysine119 ubiquitination (uH2A), respectively, to decorate the Xi by a series of repressive epigenetic modifications [15]. Among various chromatin modifying enzymes recruited to the Xi in a Xist RNA-dependent manner, ASH2L is unique because it is known as a component of the MLL/SET histone 3 lysine 4 (H3K4) methyltransferase complexes for transcriptional activation [16,17]. To date, the function of ASH2L on the Xi remains mysterious.

*Xist* evolved from a protein-coding gene *Lnx3* in early eutherians by pseudogenization, including the integration of mobile elements which yield simple tandem repeats within *Xist* (denoted as repeat A-F) [18,19]. These Xist repeat elements play various important roles to fulfill the unique function of *Xist* during XCI [9,10,20]. Xist repeat A is required for induction of X-linked gene silencing and Xist RNA spreading across the Xi [21,22]. A recent study identified the repeat A binding proteins as potential candidates responsible for induction of the Xist repeat A-dependent X-linked gene silencing [23]. The region across the Xist repeat F and B has an important role to recruit PRC2 to the Xi [24]. Interference using peptide nucleic acid (PNA) or locked nucleic acid (LNA) complementary to Xist repeat C revealed that the repeat C is important for Xist RNA localization [25,26]. In contrast to the intensely-studied Xist exon 1 which contains the repeat A-D and F, exon 7 of *Xist* has been overlooked even though it is the second largest exon with the repeat E element highly conserved among the eutherian mammals [27]. Although *Xist* exon 7 interacts with hnRNP U and is required for the stable Xist RNA localization to the Xi [28], the role of the *Xist* repeat E localized at the 5′-end of *Xist* exon 7 has not yet been addressed. Here, we explore the link between ASH2L and Xist RNA and demonstrate the role of the Xist repeat E for ASH2L recruitment to the Xi, regulation of histone modifications and escape gene expression.

## Results

### ASH2L preferentially binds to the repeat E of Xist RNA

**Fig 1:**
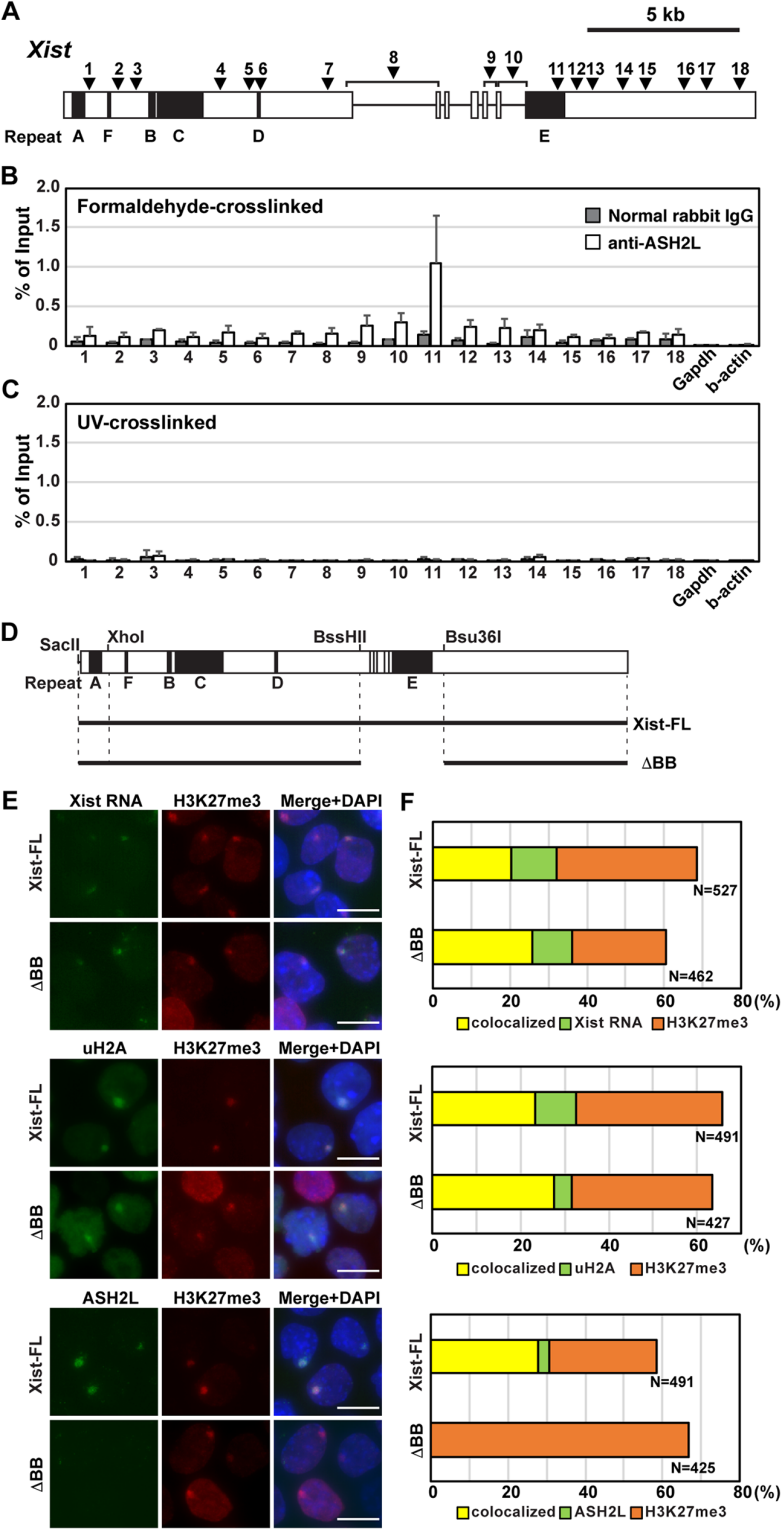
ASH2L recruitment to the Xi depends on the repeat E of Xist RNA. (A) Map of primer pairs across *Xist* for the RIP analysis. White boxes indicate *Xist* exons. The *Xist* repeats A-F are shown by black boxes. The positions of the primer pairs across *Xist* are shown as arrowheads. (B) Formaldehyde-crosslinked Xist RIP using anti-ASH2L antibody. A relative amount of Xist RNA immunoprecipitated by anti-ASH2L antibody to input was quantified by RT-qPCR. The means ± standard deviation (SD) from three independent experiments are shown. (C) UV-crosslinked Xist RIP using anti-ASH2L antibody. The mean ± SD from three independent experiments is shown. (D) Schematics of tet-inducible full-length and mutant Xist cDNAs used in this study. (E) ImmunoFISH and immunostaining of the tet-inducible *Xist* T20 ES cells at day 2 in the presence of Dox. Nuclei were counterstained by DAPI. Scale bar is 5 μm. (F) Summary of the tet-inducible mutant *Xist* cDNA experiment. Colocalization percentage was calculated by immunoFISH and immunostaining experiments in Fig 1E. More than 100 nuclei for each transgenic ES cell line in more than two independent experiments were counted.

To explore the interaction between ASH2L and Xist RNA, we carried out formaldehyde-crosslinking RNA immunoprecipitations (RIPs) (Fig 1 and S1 Fig). *Tsix*-mutant female ES cells in which non-random XCI is induced in the *Tsix*-mutant X-chromosome were differentiated, and cell lysate for the RIP was prepared at day 8 upon differentiation [29]. While the control RIP assay using normal rabbit IgG did not show significant immunoprecipitation of the Xist RNA by any primer pairs for quantitative RT-PCR (RT-qPCR) across *Xist*, Xist RNA was immunoprecipitated by anti-ASH2L antibody. The ASH2L binding to Xist RNA was especially evident at the primer pair 11, which amplifies the internal region of the repeat E. RT-qPCR using primer pairs around the repeat E (positions 9 and 13) showed modest peaks, suggesting that ASH2L preferentially binds to the repeat E region of Xist RNA (Fig 1B). Interestingly, UV-crosslinking RIP to address the direct protein-RNA interaction did not show significant precipitation of Xist RNA at any positions even with the anti-ASH2L antibody (Fig 1C). These data suggest that ASH2L could bind to the repeat E region of Xist RNA indirectly via another Xist RNA-associated protein(s). Alternatively, Xist RNA-ASH2L interaction might be too weak or transient to be detected by UV-crosslinked RIP due to its inefficient crosslinking.

To test whether the repeat E of Xist RNA is essential for ASH2L recruitment to the Xi, we used a tetracycline (tet)-inducible *Xist* transgene system with the *Xist* mutant transgenes lacking a BssHII-Bsu36I 2.6-kb region including repeat E [21]. The *Xist*-inducible male ES cell lines carry *Xist* cDNA transgene under the control of a tet-inducible promoter in the *Hprt* locus. Robust full-length *Xist* (Xist-FL) expression induced by adding doxycycline (Dox) in the culture medium resulted in *cis*-accumulation of Xist RNA called Xist RNA clouds; these Xist RNA clouds were co-localized with strong focal ASH2L signal as well as repressive epigenetic modifications, H3K27me3 and uH2A (Fig 1D–1F). On the other hand, induction of Xist RNA lacking the repeat E (Xist-BB) failed to recruit ASH2L, although localization of Xist RNA, H3K27me3 and uH2A to the Xi was not altered (Fig 1D–1F). These results suggest that 2.6-kb region containing repeat E is crucial for ASH2L recruitment to the Xi and that Xist repeat E and ASH2L are not required for the *cis*-accumulation of Xist RNA, H3K27me3 and uH2A on the Xi.

### Xist repeat E deletion induces slightly increased *Xist* expression

**Fig 2:**
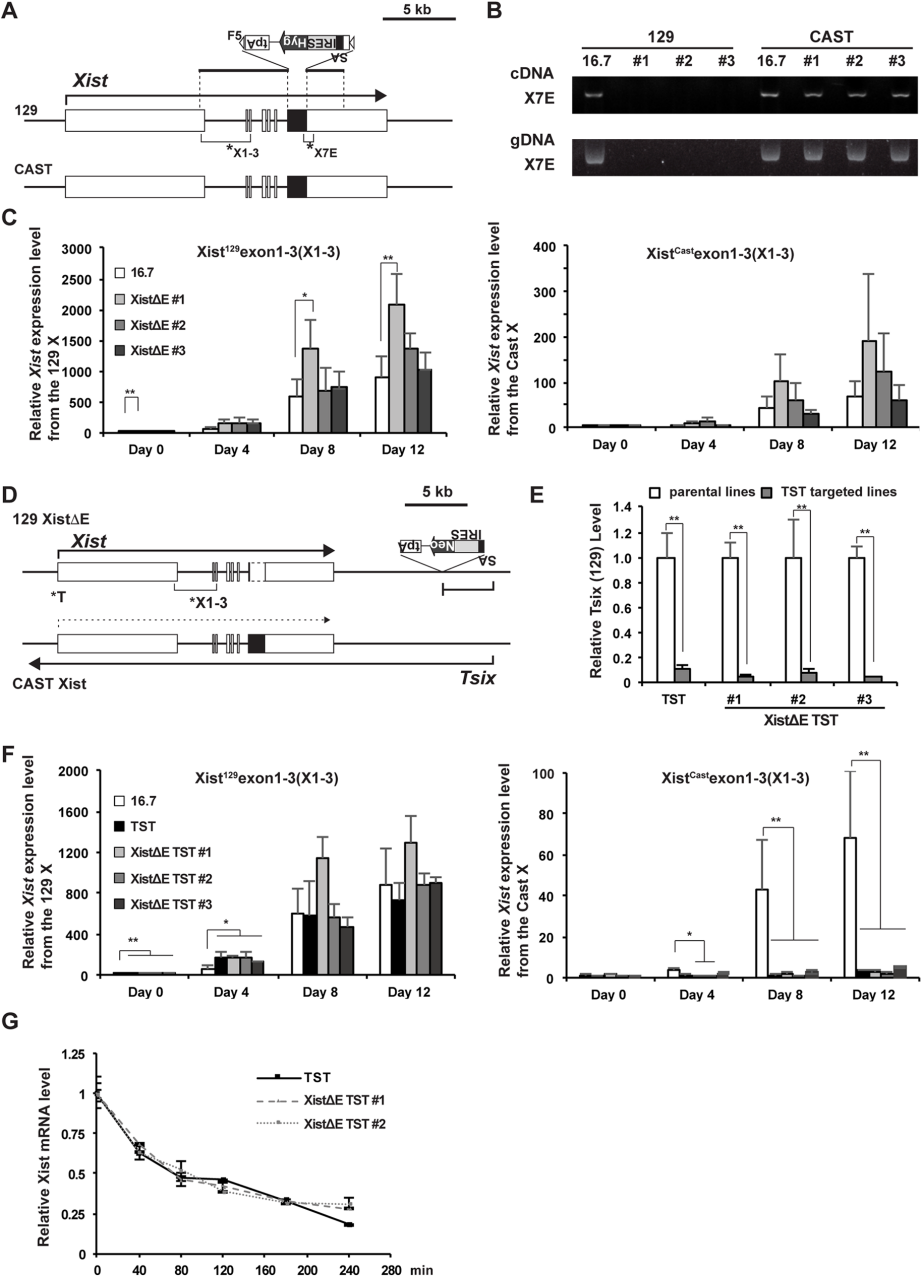
Xist induction occurs normally in the Xist repeat E mutant female ES cells upon differentiation. (A) A map of *Xist*/*Tsix* locus to show the targeted deletion of the *Xist* repeat E (XistΔrepE) on 129 allele. The targeting vector is shown above the map. SA, Splice acceptor; IRES, internal ribosomal entry site; Hyg, hygromycin resistance gene; tpA, tandem polyadenylation signals. The positions of primer pairs used for allele-specific *Xist* expression analysis (B and C) are shown as asterisks. (B) Allele-specific RT-PCR and genomic PCR analysis for *Xist* using the primer pair (X7E) which amplifies the 3′-end of the Xist repeat E in the XistΔrepE mutant cell lines. (C) Allele-specific RT-qPCR of the *Xist* expression across exon 1–3 (X1-3) in the XistΔrepE mutant. Gapdh was used as an internal control for normalization. Each value was also normalized to that of the undifferentiated wildtype 16.7 cells which is set to 1. The mean ± SD from three independent experiments is shown. *P*-values were calculated by an unpaired t-test (*p<0.05, **p<0.01). (D) A map of *Xist*/*Tsix* locus to show the targeted truncation of the *Tsix* in XistΔrepE cells on 129 allele. The positions of primer pairs used for allele-specific *Tsix* and *Xist* expression analysis (E and F) are shown as asterisks. (E) Allele-specific RT-qPCR of the *Tsix* expression at exon 4 (T) in the XistΔrepE /TST mutants. Gapdh was used as an internal control for normalization. The expression values were normalized to Gapdh and those of the parental cells. (F) Allele-specific RT-qPCR of the *Xist* expression across exon 1–3 (X1-3) in the XistΔrepE mutant. The expression values were normalized to Gapdh and those of the undifferentiated wildtype 16.7 cells (D). The mean ± SD from three independent experiments is shown. (G) Half-life assay for Xist RNA in the TST and XistΔrepE/TST mutant cells. The mean ± SD from two independent experiments is shown.

To further examine the role of *Xist* repeat E for ASH2L recruitment to the Xi and random XCI, we created *Xist* repeat E deletion mutant (XistΔE) female ES cells using standard gene targeting (Fig 2A and S2 Fig). It is likely that deletion of a specific region of Xist RNA which interacts with ASH2L could disrupt ASH2L function on the Xi but not affect global transcriptional activation by the MLL/SET complexes. To delete the 1.3 kb repeat E region at the 5′-end of *Xist* exon 7, we replaced the repeat E by a hygromycin resistant gene (Hyg) cassette flanked with two FRT derivative F5 sites (Fig 2A and S2A Fig). Using genomic PCR screening to detect the targeted insertion of the selection cassette at the Xist repeat E, we identified several XistΔE female ES cell lines (S2B Fig). The SA-IRES-Hyg-tpA cassette was finally removed by Flpe expression in the targeted XistΔE ES clones (S2C and S2D Fig).

Parental mouse hybrid female ES cell line 16.7 carries an X chromosome from *Mus musculus* 129SvJ (129) and the other from *Mus castaneus* (Cast) [30]. In this cell line, approximately 70–80% of the Xi is derived from the 129 allele due to the effect of an allele-specific X-controlling element (Xce) which controls probability of random XCI [31]. Since homology arms in the targeting construct were created from the phage P1 clone [32], which has the 129 genomic fragment, it was expected that targeted deletion of the repeat E element occurred on the 129 allele in the 16.7 female ES cells. To address whether the targeting of Xist repeat E occurs on the 129 or Cast allele, we carried out allele-specific RT-qPCR analysis using a primer pair to amplify an internal part of Xist repeat E. Since 129 allele-specific primer pairs could not amplify the fragment from both cDNA and genomic DNA of the mutant ES cells (Fig 2B), we concluded that the *Xist* repeat E deletion occurred on the 129 allele.

To address whether the XistΔE mutation affects *Xist* expression during random XCI, random XCI was induced upon differentiation by depletion of LIF and 2i inhibitors from the culture media. Using an allele-specific primer pair across *Xist* exon 1–3, we performed RT-qPCR analysis for *Xist* using the differentiating XistΔE cells (Fig 2C). The allele-specific RT-qPCR analysis for *Xist* showed that *Xist* expression was gradually increased from both 129 and Cast alleles upon induction of XCI in both wildtype and the XistΔE mutant cells. Interestingly, we found that the expression of *Xist* changed in three independent XistΔE mutant ES clones compared with that of wildtype cells. At the undifferentiated stage, all mutant cells showed a higher *Xist* expression level compared with wildtype. In the differentiating stages, all XistΔE mutant clones showed a higher *Xist* expression level than wildtype, although the extent of *Xist* upregulation varied between the mutant ES clones. These data indicate that repeat E might affect *Xist* expression.

To clarify the role of Xist repeat E for ASH2L recruitment and X-linked gene expression on the Xi during random XCI, we introduced an additional mutation in *Tsix*, an antagonist for *Xist*, to the XistΔE mutant 129 X-chromosome, which leads to non-random XCI of the XistΔE/*Tsix*-mutant 129 X-chromosome (Fig 2D and S3A Fig) [30,33]. As a control, we used the *Tsix* mutant female ES cells. To address whether the *Tsix* expression is disrupted by the targeted truncation (*Tsix* truncation mutation, TST), we performed allele-specific RT-qPCR analysis for *Tsix* and confirmed that the expression level of *Tsix* was significantly reduced in all TST and TST/XistΔE mutant cells compared with their parental cell lines (Fig 2E). Next, to examine whether the XistΔE mutation affects XCI, we induced XCI upon ex vivo differentiation using the TST and TST/XistΔE mutant cell lines. The growth of the TST/XistΔE cells upon differentiation was normal and could not be distinguished from the control TST cells (S3B Fig).

To confirm non-random XCI induced by the *Tsix* mutation, we performed the RT-qPCR analysis for *Xist* and confirmed non-random expression of *Xist* from the 129 allele (Fig 2F). As a result of the *Tsix* mutation, robust *Xist* upregulation from the 129 allele occurred at an earlier time point upon differentiation in the TST and TST/XistΔE mutant cell lines than in the wildtype cells. On the contrary, *Xist* expression from the Cast active X-chromosome (Xa) was significantly repressed. Similar to the slightly increased *Xist* expression observed in the XistΔE mutant cell lines, a slightly higher *Xist* expression level was observed in the TST/XistΔE mutant cell lines compared to the control TST cells.

To clarify whether the slightly higher *Xist* upregulation in the XistΔE mutant cell lines is a result of altered Xist RNA stability, we carried out the half-life assay for Xist transcript (Fig 2G). After pulse-labeling of nascent transcripts by 5-ethynyl uridine (EU) for 24 hours, the EU-labeled cells cultured in normal medium were retrieved every 40 minutes. EU-labeled RNA clicked by biotin was captured by streptavidin beads and compared with the INPUT RNA by RT-qPCR analysis using a Xist RNA-specific primer pair. The half-life of Xist RNA was approximately 80 minutes and did not significantly differ between the TST and TST/XistΔE mutant cells. This suggested that the upregulation of *Xist* expression observed in the XistΔE mutant cells might be a result of increased transcription, not alteration of its stability.

### The XistΔE mutation abrogates ASH2L recruitment to the Xi but does not affect the deposition of repressive histone modifications

**Fig 3:**
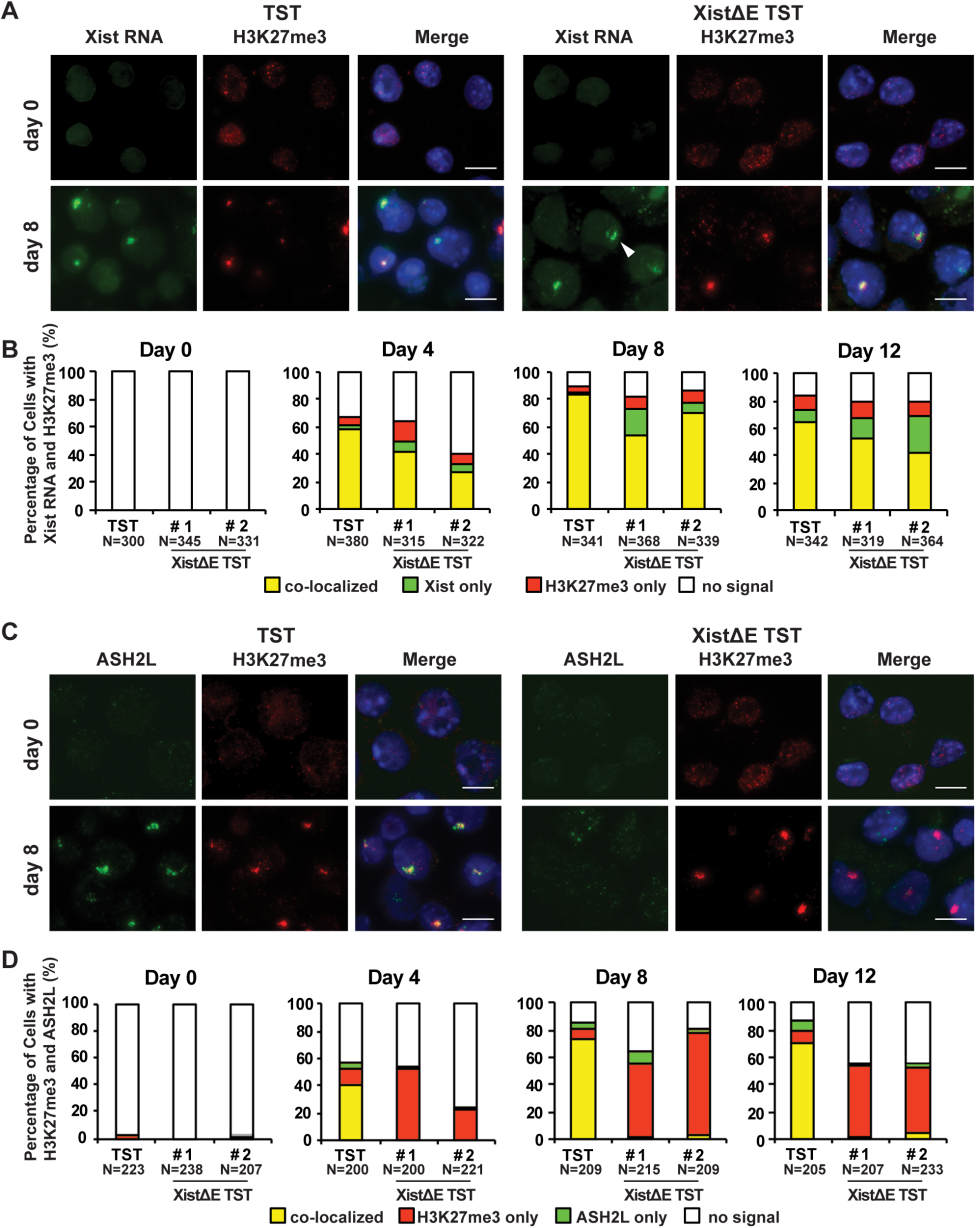
The repeat E of Xist RNA is required for the loading of ASH2L to the Xi but not for the deposition of the repressive epigenetic modifications. (A) Immuno-FISH for Xist RNA (green) and H3K27me3 (red) at day 0 and 8 upon differentiation. Nuclei were counterstained by DAPI. Arrowhead indicates typical dispersed Xist RNA cloud. Scale bar is 10 μm. (B) Frequency of Xist RNA cloud and H3K27me3 positive cells during differentiation from three independent experiments. More than 300 nuclei in each ES cell line at each time point were counted. (C) Immunostaining for ASH2L (green) and H3K27me3 (red) at day 0 and 8 upon differentiation. Nuclei were counterstained by DAPI. (D) Co-localization frequency of H3K27me3 and ASH2L from two independent experiments. More than 200 nuclei in each ES cell lines at each differentiation time were counted.

During XCI, Xist RNAs cover the entire Xi and recruit various chromatin modifying enzymes to the Xi to induce chromosome-wide dynamic epigenetic alteration. To determine whether the XistΔE mutation affects Xist RNA localization and H3K27me3 deposition on the Xi, we performed the Xist RNA florescence in situ hybridization (FISH) combined with immunofluorescence with an anti-H3K27me3 antibody (Fig 3A and 3B). Neither Xist RNA clouds nor H3K27me3 strong foci could be seen in undifferentiated ES cells. As differentiation progressed, the co-localization of Xist RNA and H3K27me3 was gradually increased in both control TST and XistΔE TST cells. Interestingly, we observed dispersed Xist RNA localization in a subset of the XistΔE TST cells although H3K27me3 signal was focal and colocalized with the Xist RNA clouds.

We also examined the localization of other repressive epigenetic histone modifications, uH2A and H4K20me1, which are recruited to the Xi upon induction of random XCI [34–36]. Focal staining of these repressive epigenetic marks was colocalized with H3K27me3 and was not altered by the repeat E deletion (S4 Fig). Although the deposition of H3K27me3 and uH2A on the Xi was not affected by *Xist* repeat E deletion, we also examined the recruitment of EZH2 and RING1B, the catalytic core subunits of PRC2 and PRC1 complex, respectively, by immunoFISH (S5 Fig). At ES cell stage (day 0), neither Xist RNA cloud nor EZH2/RING1B focal signal was observed in both TST and XistΔE TST cells. As differentiation progressed, EZH2 and RING1B showed the highest colocalization with Xist RNA cloud at day 4. The percentage of their colocalization gradually decreased at day 8 and 12 in both TST and XistΔE TST cells as reported previously [35–37]. These results demonstrated that the Xist repeat E is not required for the recruitment of some repressive epigenetic histone modifications to the Xi. We also performed immunostaining analysis using anti-H3K27me3 and ASH2L antibodies and confirmed that the deletion of the repeat E resulted in the depletion of ASH2L from the Xi. Since the other common components of the MLL/SET complexes, WDR5 and RBBP5, were not enriched on the Xi upon the induction of random XCI (S6 Fig), function of ASH2L —which is recruited to the entire region of the Xi by Xist RNA—would be independent of the MLL/SET complexes' activity. Taken together, these data suggest that Xist repeat E is essential for the ASH2L localization but not for the deposition of repressive epigenetic modifications on the Xi.

### Escape gene expression is compromised in the XistΔE mutant cells

**Fig 4:**
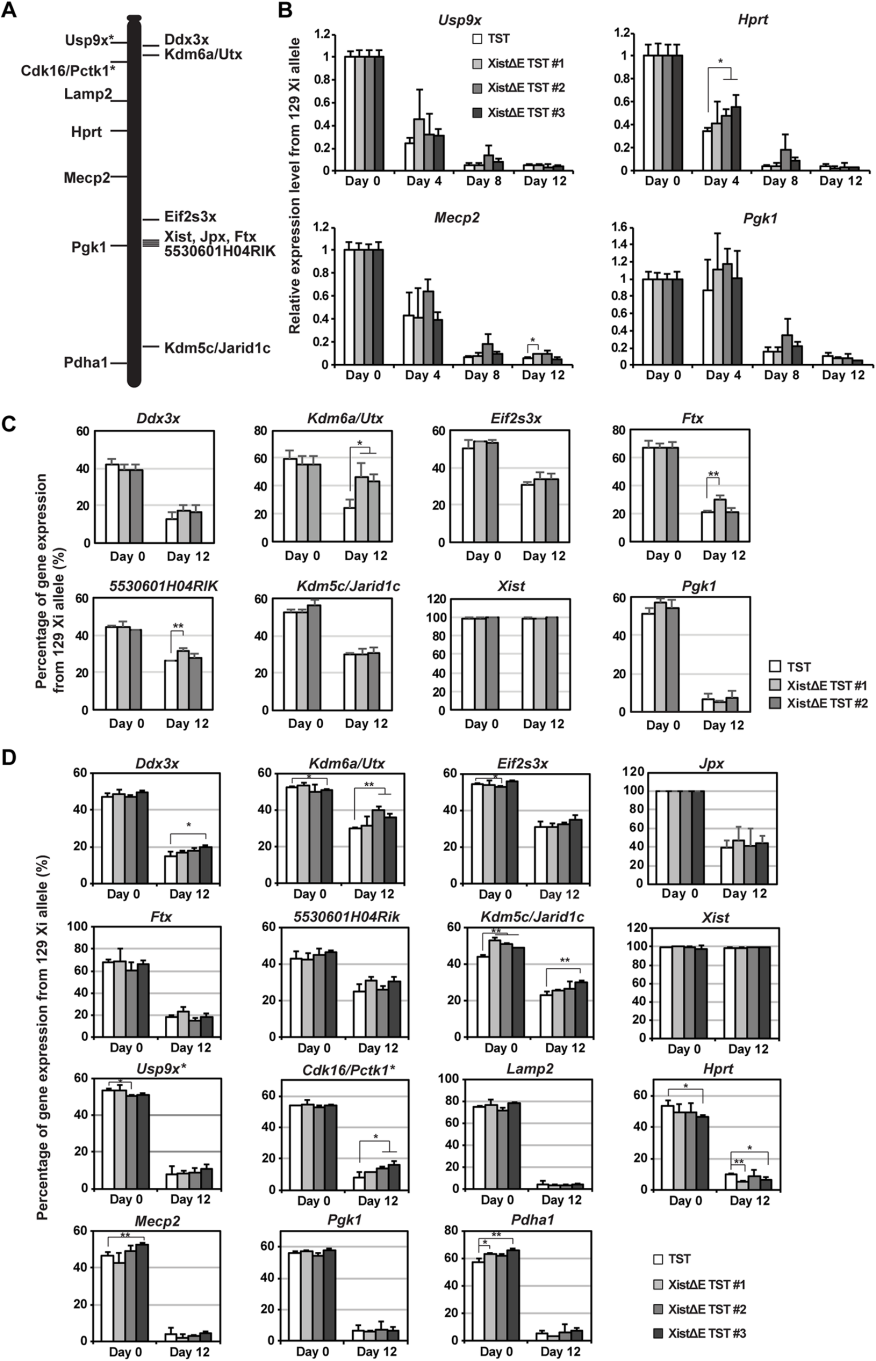
The Xist repeat E deletion alters escape gene expression but not affect X-linked gene silencing. (A) Location of non-escape (left) and escape genes (right) on the X-chromosome we tested in Fig 4B–4D. Astarisk indicates a gene known as an escape gene in brain. (B) 129 mutant-allele-specific RT-qPCR analysis of X-linked *Pgk1* and *Mecp2*. The data was normalized to Gapdh and undifferentiated TST cells (set to 1). The mean ± SD from three independent experiments is shown. *P*-values were calculated by an unpaired t-test (*p<0.05). (C) Allele-specific expression analysis of X-linked genes using pyrosequencing. The allelic ratio of expression for each sample was normalized to pyrosequencing data using genomic PCR fragment (X^129^:X^Cast^ = 50%:50%). The mean ± SD from three independent experiments is shown. *P*-values were derived from an unpaired t-test (*p<0.05, **p<0.01). (D) Allele-specific RNAseq analysis using 15 X-linked gene-specific primer sets. The mean ± SD from three independent experiments is shown. Sequencing reads matching the sequences for each transcript/allele (129 or Cast) were counted by a custom python script. *P*-values were derived from an unpaired t-test (*p<0.05).

Since the XistΔE mutation resulted in the slight upregulation of *Xist* expression and dispersed Xist RNA in a subset of differentiating cells, we next addressed whether the expression levels of X-linked genes are affected during XCI. First, we examined the expression pattern of four X-linked non-escape genes using the allele-specific RT-qPCR analysis (Fig 4A and 4B). Upon differentiation, these X-linked genes on the 129 Xi were gradually silenced and efficiently repressed less than 10% (at day 12) of that in undifferentiated cells (at day 0). Similar to the observation of in vivo X-linked gene silencing during imprinted XCI [38], each X-linked non-escape gene exhibited a diverse kinetics of gene silencing upon differentiation. Whereas *Hprt* and *Usp9x* were silenced by day 4 upon differentiation, *Pgk1* silencing started after day 4 of differentiation. While *Hprt* silencing was slightly slower in two of three XistΔE/TST mutant clones at day 4 upon differentiation, all four non-escape genes were efficiently silenced as differentiation progressed. Combined with immunostaining data in Fig 3, these results indicate that the repeat E deletion does not affect the induction of X-linked genes silencing on the Xi despite of dispersed Xist RNA clouds in a subset of the XistΔE/TST cells.

The MLL/SET complexes generally induce open chromatin formation and transcriptional activation by the deposition of methylation at H3K4 [17]; thus, we next examined whether depletion of ASH2L, a component of the MLL/SET complexes, from the Xi by the Xist repeat E deletion affects the expression of escape genes. Due to fewer single-nucleotide polymorphisms (SNPs) in the exon regions than in the intron and intergenic regions, a limited number of escape genes have been identified by various methods, including allele-specific RNAseq in mice, to date [8]. To further examine the expression ratio of escape genes between the Xa and Xi, we picked 8 X-linked genes (1 non-escape and 6 escape genes included) and addressed the impact of the *Xist* repeat E deletion on the escape gene expression using pyrosequencing analysis (Fig 4C). We confirmed dominant *Xist* expression and *Pgk1* silencing on the 129 mutant X as a result of non-random XCI of the 129 X due to the *Tsix* mutation, which is consistent with the data in Figs 2F and 4B. In undifferentiated cells, escape gene expression in the XistΔE/TST mutation cells was comparable to that in the control TST cells. Upon differentiation, however, we observed the stochastic upregulation in a subset of escape genes on the Xi. To our surprise, three out of six escape genes’ expression from Xi in the XistΔE mutation cells exhibited higher than control cells. *Utx* and *Ftx* expression was most affected by the *Xist* repeat E deletion, and the upregulation on the Xi increased to 63% compared to control cells expressing wildtype *Xist*. To further address X-linked gene expression, we performed allele-specific RNAseq analysis using 15 X-linked gene-specific primer sets (Fig 4D). Similar to pyrosequencing data, a subset of escape genes (3 out of 8 escape genes tested) was upregulated in some clones while expression of all 5 non-escape genes was stably silenced in the XistΔE/TST cells. *Cdk16/Pctk1* and *Usp9x*, known escape genes in the brain, showed slightly higher expression than typical non-escape genes (Fig 4D) [39]; thus, they might be escape genes in female ES cells upon differentiation. Since escape gene expression ratio between the Xa and Xi was not altered between the TST and XistΔE/TST mutation cells in undifferentiated cells, deletion of *Xist* repeat E results in compromised escape gene expression in a differentiation-specific manner.

**Fig 5:**
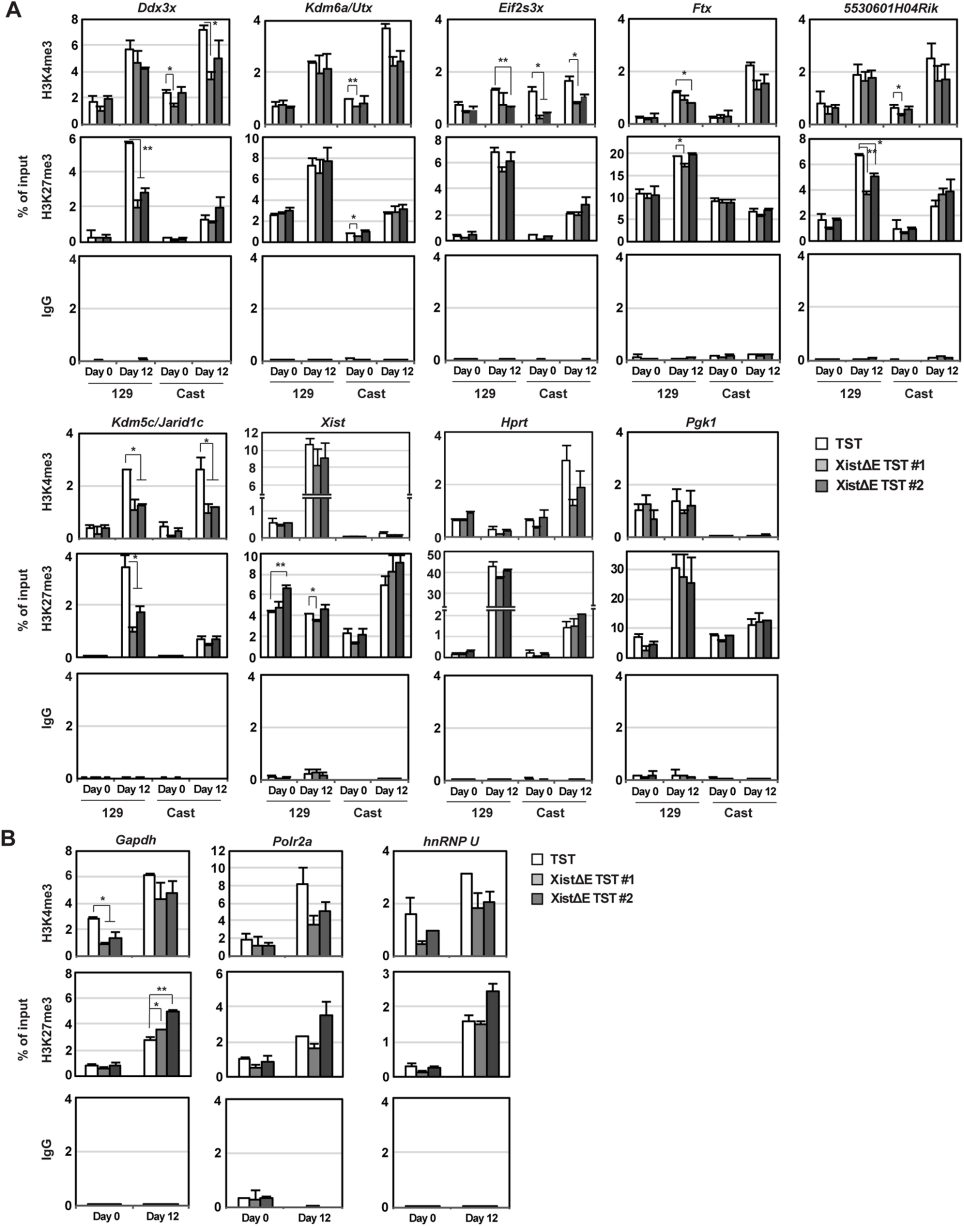
H3K27me3 level at X-linked gene promoter is reduced on the Xi in XistΔE mutant. (A) Allele-specific ChIP experiments of X-linked genes with antibodies against H3K4me3, H3K27me3, and control mouse IgG. The mean ± SD from two independent experiments is shown. Statistical significance was calucuated by an unpaired t-test (*p<0.05, **p<0.01). (B) ChIP experiments of autosomal genes with antibodies against H3K4me3, H3K27me3, and control mouse IgG. The mean ± SD from two independent experiments is shown. *P*-values were derived from an unpaired t-test (*p<0.05, **p<0.01).

To explore a potential link between epigenetic alteration and higher escape gene expression induced by ASH2L depletion from the Xi, we performed allele-specific chromation immunoprecipatition (ChIP) using anti-H3K4me3 and -H3K27me3 antibodies (Fig 5). As previously reported [40], H3K4me3 levels at X-linked gene promoter in the control TST cells (Fig 5A, white columns) were relatively comparable between the Cast Xa and 129 Xi even at day 12 upon differentiation, indicating that H3K4me3 might not be a major epigenetic regulator for X-linked gene silencing at the onset of random XCI. In contrast, H3K27me3 levels at the 129 Xi promoters in the control TST cells were higher than at the Cast Xa upon differentiation due to PRC2 recruitment to the Xi. Surprisingly, H3K4me3 levels at the promoter region of X-linked genes were significantly reduced on both Xa and Xi in the XistΔE/TST cells especially upon differentiation. XistΔE/TST ES cells (day 0) exhibited largely similar level of H3K4me3 at X-linked gene promoters, suggesting Xist RNA-dependent ASH2L on the Xi might regulate a chromosome-wide H3K4me3 distribution. Since H3K4me3 levels at escape gene promoters in the XistΔE/TST cells at day 12 upon differentiation were comparable between the Xa and Xi (Fig 5A), H3K4me3 is unlikely to cause upregulation of escape gene on the Xi in the XistΔE/TST cells upon differentiation. Interestingly, H3K4me3 at autosomal gene promoters was also affected by the *Xist* repeat E deletion (Fig 5B), suggesting that *Xist* repeat E might regulate global H3K4me3 distribution in female cells. Since H3K27me3 demethylase *Utx* expression was one of the escape genes which exhibited upregulated expression in the XistΔrepE cells, we next addressed whether reduction of H3K27me3 level at the promoter region is associated with abnormal upregulation of a subset of escape gene in the XistΔrepE/TST cells by allele-specific ChIP. Interestingly, the H3K27me3 modification at the promoter regions on the Xi significantly declined only in a subset of genes (*Ddx3x*, *5530601H04Rik* and *Kdm5c/Jarid1c*) in the XistΔrepE cells, although EZH2 recruitment and H3K27me3 deposition to the Xi during EB differentiation were indistinguishable between control TST and XistΔE/TST cells (Figs 3 and S5), suggesting that the reduction of H3K27me3 levels at some escape gene promoters might cause stochastic upregulation of escape genes on the Xi. Since reduction of H3K27me3 occurred only in a subset of genes and not on a global level, *Utx* upregulation might not cause escape gene upregulation in the XistΔE/TST cells. In sum, these results demonstrate that *Xist* repeat E is essential for the establishment of global H3K4me3 distribution and H3K27me3 modifications at the escape gene promoter regions on the Xi.

## Discussion

In this study, we have identified the repeat E of Xist RNA as an essential region for ASH2L recruitment to the Xi by a formaldehyde crosslinked RIP experiment and targeted deletion. In our recent study, direct interaction between hnRNP U and Xist RNA was revealed using a UV-crosslinked RIP[28]. Interestingly, the repeat E region is the least preferential binding region for hnRNP U, which preferentially binds to exon 7 of Xist RNA where the repeat E resides at the 5′ end. The repeat E region could have a distinct function from those required for Xist RNA localization on the Xi via hnRNP U, which has both DNA and RNA binding domains at N- and C-terminals, respectively [41,42]. Recent work attempted to uncover the conformation of full-length Xist RNA in vivo [43–45]. Whereas the repeat E in Xist RNA is predicted as an unstructured region using computational analysis, in vivo dimethyl sulfate (DMS) chemical probing analysis indicated that the repeat E of Xist RNA is protected from the DMS attack, implying that the repeat E region could be protected by protein-RNA interactions or by forming a paired secondary structure [43,45]. More recent analysis for global RNA duplex formation based on reversible psoralen crosslinking (PARIS) enables us to clarify base-pairing interactions on an individual-molecular level [44]. PARIS analysis using human HEK293 cells indicates that the frequent local and long-range helix formation within XIST RNA exon 6 (a counterpart of *Xist* exon 7 in mice) occurs where the repeat E resides. Since this domain structure of RNA duplex formation across XIST exon 6 is conserved, it is possible that unique duplex folding including the repeat E might be recognized by a variety of chromatin regulators including ASH2L, such as XIST RNA repeat A-SPEN RNA-protein complex [23,44]. While ASH2L interacts with Xist RNA repeat E, ASH2L also has DNA-binding activity at its N-terminal forkhead-like helix-wing-helix domain [46]. Putative DNA-binding affinity of ASH2L and its association with Xist RNA indicate that ASH2L might play an important role to construct a unique conformational structure of the Xi during XCI and may be involved in epigenetic dynamics on the Xi and escape gene expression.

Although we have identified the repeat E of Xist RNA as a binding region of ASH2L, a recent formaldehyde-crosslinked RIP analysis using a human myelogenous leukemia cell line revealed that several chromatin-associated proteins, such as components of PRC2 (SUZ12 and EZH2) and a component of histone deacetylase NuRD complex (CHD4), also bind to repeat E [47–49]. Our data in Figs 3, 4 and S5, however, indicate that the repeat E is dispensable for X-linked non-escape gene silencing and for the recruitment of EZH2 and H3K27me3; thus, *Xist* repeat E is unlikely to be a major EZH2 binding region observed in a human myelogenous leukemia cell line. Indeed, localization of PRC2 on the Xi is transient during ES cell differentiation (S5 Fig) [37]. H3K27me3 modification on the Xi is proposed to be maintained by the association with the perinucleolar region in differentiated cells [50]. Thus, the role of the repeat E of Xist RNA as a scaffold for chromatin modifying factor might differ in the context of cell type. However, we found that the H3K27me3 level was downregulated at the promoter in a subset of escape genes on the XistΔE Xi (Fig 5). Although the molecular mechanism underlying H3k27me3 modification at the escape gene promoters remains to be elucidated, Xist repeat E and ASH2L could be involved in the H3K27me3 distribution at the escape gene promoters on the Xi.

Surprisingly, the depletion of ASH2L from the Xi by the repeat E deletion resulted in transcriptional upregulation of a subset of escape genes we tested without affecting stable silencing of non-escape genes during XCI. We initially hypothesized that escape gene expression on the Xi is repressed due to the depletion of ASH2L from the Xi, since ASH2L is a common component of the MLL/SET H3K4 methyltransferase complexes for transcriptional activation [17]. As shown in our results and previous report [16], other common components of the MLL/SET complexes such as WDR5 and RBBP5 are not enriched on the Xi during XCI, suggesting a unique role for ASH2L on the Xi. Because of the enrichment of H3K4me2 and 3 at escape gene loci [40,51], we cannot eliminate the possibility that ASH2L is required locally for escape gene expression on the Xi as a part of the MLL/SET complexes. It is also interesting that Xist repeat E deletion affects H3K4me3 levels at autosomal and X-linked gene promoters (Fig 5A and 5B). The alteration of global H3K4me3 distribution might disturb global gene expression and partly contribute to an increased ratio of escape gene expression from the Xi compared to the Xa in the XistΔE/TST mutant cells (Fig 4C and 4D). Since *Xist* repeat E deletion resulted in increased free ASH2L in the nucleus, the increased amount of ASH2L might disturb the MLL/SET complexes' activity. Xist RNA might act as a molecular sponge of ASH2L to regulate the MLL/SET complexes' activity. A similar sponge function of noncoding gene *Jpx*, a positive regulator for *Xist*, has been reported [52]. *Jpx* expression is essential for *Xist* upregulation at the onset of random XCI through eviction of *Xist* repressor CTCF by its binding affinity with CTCF.

The physiological roles of Xist repeat E, of which mutations affect escape gene expression, remain to be elucidated. Several X-linked genes encoding chromatin modifying enzymes and transcriptional regulators, such as *Kdm5c/Jarid1c* and *Kdm6a/Utx* (histone demethylases for H3K4 and H3K27, respectively) and *Ddx3x* (ATP-dependent RNA helicase), are known as escape genes[8]. Recent studies have identified frequent mutations in genes involving transcriptional regulation such as chromatin modifying enzymes in human cancer, suggesting that those genes could play a major role in cancer pathogenesis, metastasis and malignancy by altering global gene expression [53]. Aberrant X-linked gene expression, including transcriptional regulators, in cancer cells has been also reported [54]. Indeed, overexpression of the escape genes *KDM5C*, *KDM6A* and *DDX3X* is associated with cancer development, proliferation, invasion and metastasis in certain type of cancers such as breast cancer [55–59]. In addition, significantly higher expression of the escape long noncoding *FTX* gene has been reported in colorectal cancer, although the downregulation of *FTX* is also associated with primary breast tumors [60,61]. *Ftx* potentially activates *Xist* in random XCI in mice [62]. Since partial upregulation of a subset of X-linked genes by *Xist* knockout in the hematopoietic stem cells leads to hematological cancer in mice [63], the strict conservation of appropriate level of X-linked gene expression including escape genes would be critical for suppression of cancer.

In conclusion, we provide evidence for the unique role of Xist repeat E for the recruitment of ASH2L and epigenetic and transcriptional regulation of X-linked genes, including escape genes. Further studies on the interaction between ASH2L and Xist RNA though the repeat E will help us to elucidate an underlying mechanism whereby expression levels of escape genes are properly regulated by Xist RNA.

## Methods

### Mouse ES cell culture

16.7 female ES cells were used to generate Xist repeat E deletion mutant [30]. ES cells were grown under standard conditions as described except for the addition of 2i inhibitors (3 μM CHIR99021 and 1 μM PD0325901, LC laboratories) [28]. *Xist* expression from tet-inducible *Xist* cDNA transgene in T20 cell lines was induced by adding 1 μg/ml doxycycline (Dox) in the culture medium for differentiation for 2 days [21]. Cre-LoxP mediated insertion of the inducible *Xist* cDNA transgenes was done as described [21].

### Generation of *Xist* repeat E deletion and *Tsix*-truncation mutant ES cells

We used bacterial homologous recombination system to construct the Xist repeat E targeting vector [64]. Left (delEx7-L50-F, TCGAGTTACCCTCTTTCTGGTGGTCTTTGCTTACTATCAATCATTAGTGTGTAT and delEx7-L50-R, CGATACACACTAATGATTGATAGTAAGCAAAGACCACCAGAAAGAGGGTAAC) and right (delXrepE-F2, CGAAAGTAATCCTTTCTTGGATGTTTCTTTGTATGTACATGTGCGTGTGTGTCGACGCG and delXrepE-R2, TCGACACACACGCACATGTACATACAAAGAAACATCCAAGAAAGGATTACTTT) arm adaptors for bacterial homologous recombination were annealed and inserted into pBS-2xF5-Zeo, a derivative of pBS-2xF3-Zeo, at XhoI/ClaI and BstBI/BstXI sites, respectively[28]. SmaI/SpeI SA-Ires-Hygromycin-tpA cassette of pGEM-SAIresHygtpA was inserted at PmlI/NheI between two F5 sites, yielding the targeting vector for bacterial recombination [28]. pBS-sx16delL contains a 9.8 kb fragment from Xist exon 1 to exon 7 (chrX: 103,464,270–103,474,034 in GRCm38/mm10, UCSC genome browser) derived from the P1 clone [32] in the pBluescriptII-SK(-) with unique SalI site at the 5′-end of the *Xist* genomic fragment. pBS-sx16delL was transfected into SW106 and targeted by XhoI-SalI fragment of the bacterial targeting vector, generating Xist repeat E targeting vector for mouse ES cells. The Xist repeat E targeting vector linearized by SalI was used for the electroporation using 16.7 female ES cells.

To construct Tsix-truncation vector with Neomycin cassette, we replaced the SA-Ires-Puromycin-tpA-LoxP cassette in pSS-TsixStop by SA-Ires-Neomycin-tpA or SA-Ires-Hygromycin-tpA[29]. The *Tsix* targeting vector was linearized by SfiI for transfection into mouse ES cells.

For transfection of targeting construct into mouse ES cells, 40 μg of linearized targeting vector was used for electroporation using the Biorad GenePulser at 240 V, 500 μF setting. The transfected mouse ES cells were cultured without selection drug for 24 hours and then cultured with 250 μg/ml Hygromycin or 300 μg/ml G418 for the Xist repeat E or Tsix targeting, respectively, for 7–8 days. Isolated individual ES colonies were screened by genomic PCR using primer sets (Xist repeat E targeting: 7.2TST5-F, GTCTCAGTTGCCTTCTCCTTGCTCCCACTG and UptpA-F, CTTTCCGAGGGACACTAGGCTGACTCCAT for 5′-end and SA-R; AAACCCTGGACTACTGCGCCCTACAGATCT and 7.2TSTIn-R, TCCCAGACCTCTTCAACCTGGCTCCATCTT for 3′-end; Elimination of Hyg cassette: Xint6-LR, CTGGAGTTGCATGACTAGGCCATTTGGA and Xex7-LR, TAGTTCCCCGCTCTTGGAAGTCACAGGT; Tsix targeting: TST-F, GGAGATCGCTAAAATCCCTGCCTTATAACCAA and SA-F, AAACCCTGGACTACTGCGCCCTACAGATCT; TST-R, AATTGGATATCCCTCGCATCTACCTACTTGGAA and UptpA-F) as described in S2 and S3 Figs.

### CRISPR/Cas9-mediated targeted knock-in of the 3xFLAG tag to *Wdr5*

pSpCas9(BB)-2A-Puro (pX459V2.0, Addgene plasmid #48139) was used for CRISPR/Cas9-mediated 3xFLAG knock-in to the 5´-end of the *Wdr5* coding region as described previously [28,65]. Primers (Wdr5-CRI-F, CACCGGAGAAGAAGCCAGAGACAG and Wdr5-CRI-R, AAACCTGTCTCTGGCTTCTTCTCC) were annealed and inserted into BbsI site in pX459ver2 modified with the sgRNA^(F+E)^ mutation for higher targeting efficiency [66]. Single-stranded oligodeoxynucleotide (ssODN) Wdr5-3xFLAG-KI (TCCATTGTGACTCCCCCTTCACGGTGTCCTGCCCTGTGGGCTTCAGAGCCATGGACTACAAAGACCATGACGGTGATTATAAAGATCATGATATCGATTACAAGGATGACGATGACAAGGCCACAGAGGAGAAGAAGCCAGAGACAGAGGCTGCAAGAGCACAGCCCACTCCTTCCTCATCAGCCACACAGAGCAAGGTA) was used for the homology directed repair (HDR)-mediated FLAG knock-in. The 3xFLAG knockin at the *Wdr5* locus was confirmed by genomic PCR using primer pair (Wdr5-T7E1-F1, GGCCCCTTACTATAGAGTTCAGC and Wdr5-T7E1-R1, CCACTGTTGTGTGCTCAGAAAT) and DNA sequencing.

### RNA immunoprecipitation (RIP)

Cells at 8 days upon differentiation on 15 cm dish were used for RIP. For UV-crosslinked RIP, cells on 15 cm dish (approximately 8x10^7^ cells) were washed with ice-cold PBS and irradiated in 5 ml ice-cold PBS with 400 mJ/cm^2^ at 254nm. Cells were recovered by scraping with 1 ml RSB buffer (10 mM Tris-HCl [pH 8.0], 100 mM NaCl, 2.5 mM MgCl_2_, 35 μg/ml digitonin, 1x proteinase inhibitor [Roche], 0.1 units/ml RiboLock RNase inhibitor [Thermo Scientific]) and incubated for 5 min on ice. One quarter of cells (approximately 2x10^7^ cells) were recovered by centrifuge with 1,000 rpm for 5 min at 4°C, snap frozen in liquid nitrogen and store at -80°C until RIP was performed. For formaldehyde-crosslinked RIP, 2x10^7^ cells were incubated in 10 ml PBS/0.5% formaldehyde for 10 min at room temperature. After quenching by final 125 mM glycine for 5 min at room temperature, cells were washed twice by ice-cold PBS, frozen in liquid nitrogen and stored at -80°C until RIP was performed. The UV- or formaldehyde-crosslinked cells were suspended in 0.6 ml SDS lysis buffer (50 mM Tris-HCl [pH 8.0], 1 mM EDTA, 150 mM NaCl, 1 mM DTT, 1% SDS, 1% TritonX-100, 1x proteinase inhibitor, 0.1 unit/ml RNase inhibitor). 0.2 ml aliquots each in 1.5 ml tube was sonicated by Bioruptor (setting: strong, 30 sec on/30 sec off, 3 cycles). After centrifugation with max speed for 10 min at 4°C, the supernatant was pooled in one tube. 0.1 ml aliquot of cell lysate was diluted by 1 ml dilution buffer (50 mM Tris-HCl [pH 8.0], 1 mM EDTA, 150 mM NaCl, 1 mM DTT, 1% TritonX-100, 1x proteinase inhibitor, 0.1 unit/ml RNase inhibitor) and 100 μl was taken as an input. The diluted lysate was incubated with 5 μg of ASH2L antibody (Bethyl Laboratories, A300-107A) or normal rabbit IgG (Millipore, 12–370) at 4°C for 2 hours, and then with 25 μl of Dynabeads protein G (Life Technologies) at 4°C for 1 hour. Magnetic beads were washed twice by ice-cold washing buffer (20 mM Tris-HCl [pH 8.0], 1 mM EDTA, 150 mM NaCl, 1 mM DTT, 0.1% SDS, 1% TritonX-100, 1x proteinase inhibitor, 0.4 unit/μl RNase inhibitor). UV-crosslinked RIP samples and inputs (50 μl) were incubated with 200 μl TE, 0.5% SDS, 0.5 μg/μl Proteinase K, 0.1 unites/ml RNase inhibitor for 1 hour at 37°C. Formaldehyde-crosslinked samples and inputs (50 μl) were reverse crosslinked in rev-crosslinking buffer (1xPBS, 2% N-lauroylsarcosine, 10 mM EDTA, 5 mM DTT, 1 μg/μl Proteinase K and 0.4 unit/μl RNase inhibitor) for 1 hour at 42°C and then for 1 hour at 55°C. RNA was isolated by Tri Reagent for liquid sample (MRC) with manufacturer’s instruction. RNA was precipitated with 2 μl of GlycoBlue (Life Technologies) and suspended in 20 μl of nuclease-free water. cDNA synthesis and RT-qPCR analysis was performed as described previously except for using different primer pairs 1 (Fw, ATCGTTTGGTGCTGTGTGAG and Rv, CTGGCTCGAGAATAGCCGTA), 10 (Fw, ATTACCCTTCCCCAAAGCAG and Rv, CACACCCACAATACACACTCATT) and 11 (Fw, TTGCATGCATCCCTCTCTTT and Rv, AACAGAGAAAGTGGCCCAAG) [28].

### Quantitative allele-specific RT-PCR

Allele-specific RT-qPCR analysis using SNP-based allele-specific primers for *Xist*, *Tsix*, *Mecp2*, *Pgk1* on X^129^ or X^Cast^ was performed as described previously [28]. In addition, primer sequences used in the analysis for Xist repeat E, *Usp9x* and *Hprt* are as following: *Xist* repeat E (Forward [Fw], TGTGCTCACCCCACTTGTTC, 129 Reverse [Rv], GCTCACCTAAGCCCAAAGTAA, Cast Rv, GCTCACCTAAGCCCAAAGTAG), *Usp9x* (Fw, GAAATGGAAGAAAGCAAAGAACCA, 129 Rv, CCAGGGGAATGAGGGTATTAA, Cast Rv, CCAGGGGAATGAGGGTATTAC) and *Hprt* (Fw, GTCAACGGGGGACATAAAAG, 129 Rv, GGGAAAGCAAAGTTTGCAAT, Cast Rv, GGGAAAGCAAAGTTTGCAAC).

### Half-life assay

Half-life assay for Xist RNA was performed as described previously [28].

### Immuno-FISH

Immuno-FISH was performed as described [28,67]. Antibodies used in this study are the following: anti-H3K27me3 (#0323, MABI [1:1000 dilution] and #9733, Cell Signaling [1:1000]), anti-uH2A (#8240, Cell Signaling [1:1000]), anti-H4K20me1 (#39727, Active motif [1:5000]), anti-RBBP5 (A300-109A, Bethyl Laboratories [1:400]), anti-ASH2L (A300-107A, Bethyl Laboratories [1:400]), anti-EZH2 (#612666, BD [1:200]), anti-RING1B (#5694, Cell Signaling [1:50]) and anti-FLAG-M2 (F1804, SIGMA [1:4000]).

### Allele-specific expression analysis using pyrosequencing

To determine allelic gene expression ratio between 129 and Cast alleles, RT-PCR products amplified from cDNA were analyzed by pyrosequencing analysis by Pyromark Q96 MD (QIAGEN). The relative expression ratio between the two alleles was quantified by the Pyromark MD SNP/Genotyping software according to the manufacturer’s instructions and normalized by the pyrosequencing data obtained from PCR fragment amplified from the genomic DNA (X^129^:X^Cast^ = 50%:50%). The following primers were used for pyrosequencing experiments is shown in S1 Table.

### Allele-specific RNAseq using gene-specific primer sets for X-linked genes

To determine allelic gene expression ratio of X-linked genes between 129 and Cast alleles, RT-PCR products amplified from cDNA using gene-specific primer sets for 12 X-linked genes (12 non-escape and 12 escape genes) were sequenced by Illumina HiSeq2500. RT-PCR was performed as described previously [28]. Gene-specific primers for first strand synthesis and cDNA amplification were shown in S1 Table. Equal molar of RT-PCR products of 12 X-linked genes derived from the same sample were pooled, and the second-round PCR (v2_Ad1_noMX as a common forward primer and v2_Ad2.25—v2_Ad2.60 as reverse primers with an index) was performed to attach adaptors for index and Illumina sequencing as described previously [68]. A custom python script was used to count the reads exactly matching the sequences for each transcript/allele.

### Chromatin immunoprecipitation (ChIP)

Approximately 1x10^7^ ES cells (day 0) and differented EB cells (day 12) were crosslinked in 1% formaldehyde/PBS 10 ml at room temp for 10 min and quenched with final 125 mM glycine at room temp for 5 min. After rinse with ice-cold PBS twice and swelling buffer (25mM Hepes-KOH [pH8.0], 10mM KCl, 1.5mM MgCl_2,_ 0.1% NP-40, 1mM DTT, 1x protease inhibitor cocktail [Roche]), the fixed cells were resuspended in swelling buffer and incubated on ice for 10 min. The cells were passed through G-25 needle for 10 times, and nuclei were isolated by centrifugation at 1,000 g, for 10 min at 4°C. The nuclei were suspended in sonication buffer (50mM Tris-HCl [pH 8.0], 150mM NaCl, 1mM EDTA, 0.1% Triton X-100, 0.1% Na-deoxycholate, 1% SDS, 1x protease inhibitor cocktail, 0.5mM PMSF) at the concentration of 2x10^7^cells/ml, and divided into 300 μl aliquotes. Sonication were performed in Biorupter sonicator (Diagenode) at power high for 18 cycles, each cycle for 30 seconds on and 30 second off. Cell lysates were centrifuged by table top centrifuge with max speed at 4°C for 10 minutes. The sheared chromatin supernatant were diluted 10 times with dilution buffer (20mM Tris-HCl [pH8.0], 150mM NaCl, 1mM EDTA, 1% Triton X-100, 0.1% Na-deoxycholate, 1x protease inhibitor cocktail, 0.5mM PMSF). 50 μl of anti-mouse IgG Dynabeads (Life Technologies) were incubated with 5 μg of antibody at 4 ^o^C for 3 hours, and 1 ml diluted chromatin lysate derived from approximately 2x10^6^ cells was rotated with Dynabeads associated with antibody at 4°C overnight. Antibodies used in the ChIP experiments were anti-histone H3K4me3 (#61379, Active Motif), anti-K3K27me3 (#61017, Active Motif) and mouse IgG (#12–371, Millipore) as a control. The beads were washed with dilution buffer, wash buffer A (50mM Hepes-KOH [pH 8.0], 500mM NaCl, 1mM EDTA, 1% Triton X-100, 0.1% Na-deoxycholate, 0.1% SDS, 1x protease inhibitor cocktail, 0.5mM PMSF), buffer B (20mM Tris-HCl [pH8.0], 1mM EDTA, 250mM LiCl, 0.5% NP-40, 0.5% Na-deoxycholate, 1x protease inhibitor cocktail, 0.5mM PMSF) and TE, with each buffer twice. The precipated protein/DNA complex were eluted by 200 μl of elution buffer (50mM Tris-HCl [pH8.0], 1mM EDTA, 1% SDS, 50mM NaHCO_3_) twince and pooled. The IP and input (100 μl + 300 μl elution buffer) were reverse crossed by adding final 200mM NaCl and incubated at 65°C for 4 hours. The DNA were extracted with phenol/chloroform, precipated by ethanol, and resuspended in 100 μl TE. qRT-PCR analysis was performed as described above with primer pairs in S1 Table.

## Supporting information

S1 Fig Validation of ASH2L antibody. (A) We performed RIP experiments using ASH2L antibody (Bethyl Laboratories, A300-107A) as described in Methods. For western blot, we used anti-ASH2L (Bethyl Laboratories, A300-112A) and Protein A-HRP (Invitrogen, 10–1023). (B) We used the auxin-inducible degron (AID) system to validate the ASH2L antibody. In the presence of the auxin perceptive F-box protein OsTIR1 (E3 ubiquitin ligase), proteins fused with an AID-tag can be induced for rapid degradation by adding auxin. Female ES cells expressing OsTIR1 and minimal AID (mAID)-tagged ASH2L were created by the CRISPR/Cas9-based mAID-tagging method as described [69,70], and will be described in detail elsewhere (M. Y. and Y. O.). Degradation of mAID-tagged ASH2L was induced for 24 hours by addition of 500 μM synthetic auxin (1-Naphthaleneacetic acid [NAA], SIGMA 35745) in ES culture medium. Western blotting was performed by ASH2L antibody (Bethyl Laboratories, A300-107A) and anti-rabbit IgG HRP (Jackson ImmunoResearch, 711-035-152) as primary and secondary antibodies, respectively.
(AI) Click here for additional data file.

S2 Fig Targeted deletion of *Xist* repeat E in female ES cells. (A) Map of the mouse *Xist*. White and black boxes indicate *Xist* exons and repeat E, respectively. The targeting vector for *Xist* repeat E is shown above the map. SA, Splice acceptor; IRES, internal ribosomal entry site; Neo, neomycin resistance gene; tpA, tandem polyadenylation signals. The positions of primer pairs for genomic PCR to confirm the targeting are shown as arrows. (B) Genomic PCR analysis of wildtype (lane 1) and XistΔE (lanes 2 and 3; two independent clones, #1 and 2) female ES cells to confirm the replacement of *Xist* repeat E by the Hyg selection cassette. M, NEB 1kb DNA ladder. Black arrow heads indicate the PCR products derived from the XistΔE allele. (C) Strategy to create the XistΔE mutant. SA-IRES-Hyg-tpA cassette flanked by F5 sites were removed by the Flpe expression. (D) Genomic PCR analysis of wildtype (lane 1), XistΔE with the Hyg cassette (lanes 2 and 3; clone #1 and 2) and XistΔE without the Hyg cassette (lanes 4 and 5 which are derived from clone #1 and 2, respectively) female ES cells to confirm the deletion of Xist repeat E. White arrow head, PCR fragment derived from the wildtype allele. Black arrow head, PCR fragment derived from the XistΔE (+Hyg) allele. Grey arrow head, PCR fragment derived from the XistΔE (-Hyg) allele.
(AI) Click here for additional data file.

S3 Fig Targeting strategy of *Tsix*-truncation mutant. (A) Map of the mouse *Xist*/*Tsix* locus. White and black boxes indicate *Xist* exons and repeat E, respectively. The targeting vector for *Tsix* is shown above the map. The positions of primer pairs for genomic PCR to confirm the targeting are shown as arrows. (B) Genomic PCR analysis of wildtype (lane 1), *Tsix*-truncation (TST) mutant (lane 2) and XistΔE/TST mutant (lanes 3 and 4; two independent clones derived from the XistΔE mutant clone #1 and 2, respectively) female ES cells to confirm the *Tsix* targeting. M, NEB 1kb DNA ladder. Black arrow heads indicate the PCR products derived from the TST mutant allele. (C) Representative phase contrast images of the differentiated embryoid body (EB) cells at day 6 upon differentiation.
(AI) Click here for additional data file.

S4 Fig *Xist* repeat E is dispensable for the deposition of repressive histone modifications on the Xi. (A) Immunostaining for H3K27me3 (green) and H4K20me1 (red) at day 8 upon differentiation. Nuclei were counterstained by DAPI. Co-localization frequency of H3K27me3 and H4K20me1 at day 8 upon differentiation from three independent experiments. More than 100 nuclei in each ES cell lines at each differentiation time were counted. (B) Colocalization of H3K27me3 (green) and uH2A (red) in differentiating EB cells at day 8 of differentiation. Nuclei were counterstained by DAPI. Scale bar is 10 μm. Colocalization frequency was determined from more than two independent experiments. More than 80 nuclei in each ES cell lines at each differentiation time were counted.
(AI) Click here for additional data file.

S5 Fig Xist repE is not required for the recruitment of EZH2 and RING1B to Xi. (A) Immuno-FISH for Xist RNA (green) and EZH2 or RING1B (red) at day 0, day 4, and 12 upon differentiation. Nuclei were counterstained by DAPI. Arrowhead indicates colocalized signal of Xist RNA cloud and EZH2 or RING1B. Scale bar is 10 μm. (B) Frequency of EZH2 and RING1B positive cells colocalized with Xist RNA cloud during differentiation. More than 100 nuclei in each ES cell line at each time point were counted in two independent experiments.
(AI) Click here for additional data file.

S6 Fig WDR5 and RBBP5 are not enriched on the Xi upon the induction of random XCI. (A) Schematics of Wdr5 3xFLAG knock-in by CRISPR/Cas9. The protein coding or 5´-UTR regions are shown as a gray or white box, respectively. The 3xFLAG tag within single-stranded oligodeoxynucleotide (ssODN) is shown as a red box. The first ATG sequence of the translation start is labeled in blue. The protein coding or 5´-UTR regions are capitalized or lowercased, respectively. The sgRNA sequence and adjacent protospacer-adjacent motif (PAM) for CRISPR/Cas9 genome editing are labeled in green and red, respectively. Arrowhead indicates putative cleavage site. (B) Genomic PCR genotyping analysis of the 3xFLAG tag knock-in *Wdr5* allele. Using primers Wdr5-T7E1-F1 and Wdr5-T7E1-R1, 270 or 336 bp PCR products are amplified from the wild-type or the 3xFLAG knock-in *Wdr5* alleles, respectively. (C) Immunostaining for H3K27me3 (green) and ASH2L, RBBP5 or 3xFLAG-WDR5 (red) at day 8 upon differentiation. Nuclei were counterstained by DAPI. Scale bar is 10 μm.
(AI) Click here for additional data file.

S1 Table The list of primers used in Figs 4 and 5. (XLSX)
